# *Chb* and *nag* genes drive *N,N′-diacetylchitobiose* metabolism in probiotic *Lacticaseibacillus paracasei*

**DOI:** 10.1007/s00253-025-13656-2

**Published:** 2026-01-10

**Authors:** Víctor García-Telles, Jimmy E. Becerra, Jesús Rodríguez-Díaz, Vicente Monedero, María J. Yebra

**Affiliations:** 1 Laboratorio de Bacterias Lácticas y Probióticos, Departamento de Biotecnología de Alimentos, Instituto de Agroquímica y Tecnología de Alimentos (IATA-CSIC), Valencia, Spain; 2 Departamento de Microbiología, Facultad de Medicina, Universidad de Valencia, Valencia, Spain; 3 INCLIVA, Instituto de Investigación Sanitaría del Hospital Clínico de Valencia, Valencia, Spain; 4 Present Address: Grupo de Investigación Alimentación y Comportamiento Humano, Universidad Metropolitana, Barranquilla, Colombia

**Keywords:** *N*-glycans, Chitin, *N*,*N*′-diacetylchitobiose, *N*-acetylglucosamine, *Lacticaseibacillus*, Transcriptional regulator

## Abstract

**Abstract:**

The persistence of commensal bacteria and administered probiotics in the human gut depends to some extent on their capacity to metabolize diet and host-derived glycans. *N*,*N*′-Diacetylchitobiose (*N*-acetylglucosamine-β-1,4-*N*-acetylglucosamine; ChbNAc) is a component of *N*-glycosylated proteins and also the major degradation product of chitin. We have identified in *Lacticaseibacillus paracasei* BL23 a gene cluster, named *chb*, involved in the catabolism of ChbNAc. The cluster encodes a transcriptional regulator (ChbR), a cellobiose-type phosphoenolpyruvate-dependent sugar phosphotransferase system (PTS) IIC (ChbC), IIA (ChbA) and IIB (ChbB) components, a DUF3284-containing protein (ChbD), and a glycoside hydrolase of the newly identified GH170 family (ChbE). Inactivation of *chbC* or *chbE* prevents the growth of *L. paracasei* in ChbNAc, suggesting that the PTS is involved in its transport and phosphorylation, and that the putative hydrolase ChbE may be acting on the resulting phosphorylated ChbNAc. An *L. paracasei* mutant with inactivated *nagA*, encoding an *N*-acetylglucosamine-6P deacetylase, was also defective in ChbNAc utilization, indicating that the transformation of *N*-acetylglucosamine-6P into glucosamine-6P by NagA is necessary for ChbNAc metabolism. Transcriptional analysis showed that the *chb* genes and the *nagA* gene are regulated by substrate-specific induction mediated by the transcriptional repressors ChbR and NagR, respectively. In addition, both transcriptional regulators repressed the *nagB* gene, which encodes a glucosamine-6P deaminase that catalyzes the conversion of glucosamine-6P into the glycolytic intermediate fructose-6P. We characterized for the first time the genes responsible for ChbNAc metabolism in a member of the *Lactobacillales*. The *chb* and *nag* clusters may constitute a strategy that allows *L. paracasei* to adapt to the gastrointestinal environment.

**Key points:**

• *Lacticaseibacillus paracasei BL23 metabolizes N,N’-diacetylchitobiose*

• *The chb and nag gene clusters are involved in N,N’-diacetylchitobiose metabolism*

• *ChbR and NagR transcriptionally repressed the chb and nagAR clusters, respectively*

**Supplementary Information:**

The online version contains supplementary material available at 10.1007/s00253-025-13656-2.

## Introduction

*N*,*N*′-Diacetylchitobiose (ChbNAc) is a disaccharide of *N*-acetylglucosamine (GlcNAc-β−1,4-GlcNAc) that forms part of the core structure of the *N*-linked glycans attached to glycoproteins, which are abundant in human milk and intestinal mucosa (Yamaguchi et al. 2024). ChbNAc is also the main degradation product of chitin, one of the most widespread polysaccharides in nature, by the action of chitinases (Ren et al. 2022). The ability to utilize ChbNAc as a carbon and nitrogen source could provide certain bacterial species with an advantage in adapting and developing in competitive environments, such as the mammalian gastrointestinal tract. Given that gut microbiota play a crucial role in influencing host health (Khalil et al. 2024; Nakatsu et al. 2024), it is important to understand the regulatory mechanisms governing the catabolic pathways that enable commensal bacteria and administered probiotics to utilize carbohydrates derived from both diet and the host.

The uptake and catabolism of ChbNAc was initially studied in Gram-negative bacteria (Keyhani and Roseman 1997). *Escherichia coli* uses the phosphoenolpyruvate-dependent sugar phosphotransferase system (PTS) for transport of ChbNAc and concomitantly phosphorylates it at the C-6 position of the GlcNAc residue at the non-reducing end (Keyhani et al. 2000). The phosphorylated ChbNAc is then deacetylated and hydrolyzed into glucosamine-6P and GlcNAc (Walter et al. 2021). The metabolism of GlcNAc further required a kinase to produce GlcNAc-6P, which will be deacetylated and deaminated into fructose-6P by the action of the *N*-acetylglucosamine-6P deacetylase NagA and the glucosamine-6P deaminase/isomerase NagB, respectively. In other Gram-negative bacteria, such as *Vibrio* spp. (Kitaoku et al. 2021), and in the Gram-positive bacteria *Streptomyces coelicolor* (Iinuma et al. 2018) and *Paenibacillus* sp. (Itoh et al. 2024), ChbNAc is imported via an ABC transport system. However, the fate of the transported disaccharide varies among these species. In *Vibrio* spp., ChbNAc is hydrolyzed into GlcNAc-1P and GlcNAc by a ChbNAc phosphorylase (Hidaka et al. 2004), while in the latter bacteria, it is degraded into GlcNAc monomers by the activity of an *N*-acetyl-β-D-glucosaminidase (Itoh et al. 2019).

**Fig. 1:**
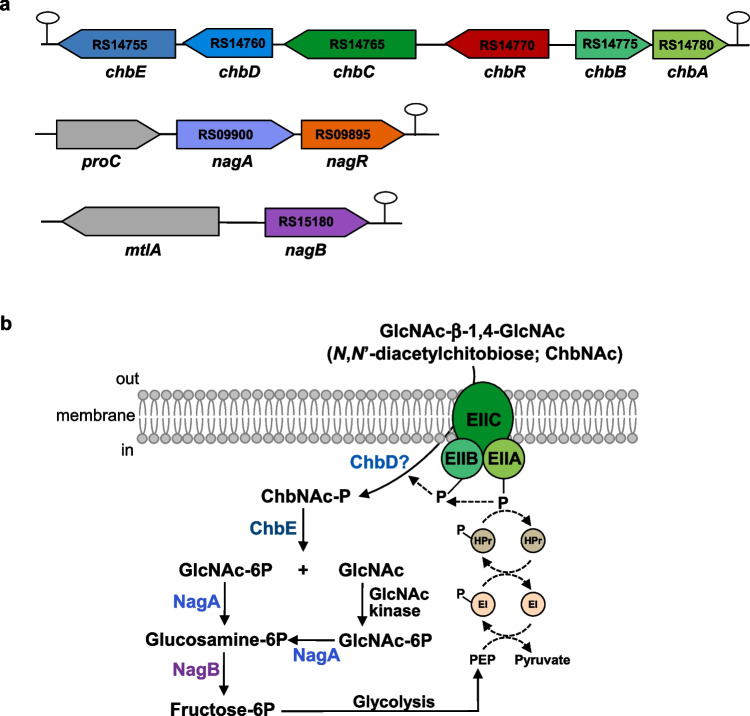
*N*,*N*′-diacetylchitobiose (ChbNAc) metabolism in *Lacticaseibacillus paracasei* BL23. **a** Structural organization of the *chb* and *nag* gene clusters in *L. paracasei* BL23. Stem-loop structures represent *rho*-independent transcriptional terminators. The letters and numbers within the arrows correspond to the ORF identification (LCABL_) from the genome sequence. **b** Schematic representationf of the transport and catabolic pathways for ChbNAc in *L. paracasei* BL23. EIIC, EIIB and EIIA, enzyme II domains of the cellobiose-type phosphoenolpyruvate:carbohydrate phosphotransferase system (PTS); HPr, Histidin-containing protein of the PTS; EI, enzyme I of the PTS; PEP, phosphoenolpyruvate; GlcNAc, *N*-acetylglucosamine; ChbD, protein containing the DUF3284 domain of unknown function; ChbE, phospho-β-*N*-acetylglucosaminidase; NagA, *N*-acetylglucosamine-6P deacetylase; NagB, glucosamine-6P deaminase

*Lacticaseibacillus paracasei* is one of the most commonly used probiotic species of lactobacilli (Hill et al. 2018). The *L. paracasei* strain BL23 has been shown to modulate the immune response (Archambaud et al. 2012; Souza et al. 2018), exhibit anti-tumoral properties (Jacouton et al. 2018) and demonstrate antimicrobial activity against pathogens (da Silva Barreira et al. 2023). This strain is able to metabolize human milk oligosaccharides, glycans derived from mucins and other glycoproteins (Bidart et al. 2014, 2018, 2016; Rodriguez-Diaz et al. 2012). The glycoamino acid fucosyl-α−1,6-*N*-GlcNAc-Asn, which is a constituent of the core-fucosylated *N*-glycans in mammals is also a substrate for *L. paracasei* (Becerra et al. 2020). We report here that *L. paracasei* BL23 is capable of fermenting ChbNAc and we identified a gene cluster, named *chb*, encoding a cellobiose-type PTS and a glycosidase required to catabolize this disaccharide (Fig. 1). The involvement of the gene *nagA*, which encodes an *N*-acetylglucosamine-6P deacetylase, in the utilization of ChbNAc by *L. paracasei* BL23 was also demonstrated. The genes *chbR* and *nagR*, which encode transcriptional regulators, were disrupted and their role in regulating the expression of the *chb* and *nag* genes was studied, showing that ChbR and NagR act as transcriptional repressors of the *chb* and *nagAR* clusters, respectively, and that both repress *nagB*, which encodes a glucosamine-6P deaminase.

## Materials and methods

### Bacterial strains, culture conditions and plasmids

The strains and plasmids used in this work are described in Table 1. *L. paracasei* strains were cultured at 37 °C under static conditions in MRS medium (Difco) for routine assays. *E. coli* DH10B and NZY5α were used as hosts in cloning experiments, and they were cultured in Luria–Bertani medium (Condalab) at 37 °C under constant agitation at 230 rpm. *Lactococcus lactis* was also used for cloning experiments and it was cultured at 30 °C under static conditions in M17 medium (Oxoid) supplemented with 0.5% glucose. The corresponding solid media were prepared by adding 1.8% agar. *E. coli* transformants were selected with ampicillin (100 µg ml^−1^) or erythromycin (100 µg ml^−1^). *L. paracasei* and *L. lactis* transformants were selected with erythromycin (5 µg ml^−1^). 

**Table 1:** Strains and plasmids used in this study

Strain or plasmid	Relevant genotype or properties	Source or reference	
**Strains**			
*Lacticaseibacillus paracasei*			
BL23	Wild type	CECT 5275	
BL372	BL23 *alfH::*pRV300 Erm^R^	(Becerra et al. 2020)	
BL388	BL23 *nagA::*pRV300 Erm^R^	(Bidart et al. 2014)	
BL402	BL23 *chbC::*pRV300 Erm^R^	This work	
BL404	BL23 *nagR* (frameshift at HindIII site)	This work	
V02	BL23 *chbE* (frameshift at NcoI site)	This work	
V03	BL23 *chbR (*112-bp internal deletion and frameshift)	This work	
V19	BL23 *chbD* (296-bp in-frame deletion)	This work	
V36	V19 containing pT1NX	This work	
V37	V19 containing pT1NchbD	This work	
*Escherichia coli*			
DH10B	F^−^ *endA1 recA1 galE15 galK16 nupG rpsL* Δl*acX74* Φ80l*acZ*ΔM15 *araD139* Δ(*ara,leu*)7697 *mcrA* Δ(*mrr*-* hsdRMS-mcrBC*) λ^−^	Invitrogen	
NZY5α	*fhuA2*Δ(*argF-lacZ*)U169 *phoA glnV44* Φ80 Δ(*lacZ*)M15 *gyrA*96 *recA*1 *relA*1 *endA*1 *thi*−1 *hsdR*17	NZYtech	
V21	NZY5α containing pQEchbR	This work	
V23	NZY5α containing pQEnagR	This work	
*Lactococcus lactis*			
MG1363	Plasmid- and phage-free derivative of NCDO712	(Gasson 1983)	
**Plasmids**			
pRV300	Suicide vector carrying Erm^R^ from pAMβ1	(Leloup et al. 1997)	
pMG36e	Editing plasmid; Erm^R^	(Gu et al. 2022)	
pRVchbE	pRV300 with a frameshift at NcoI site in *chbE* fragment	This work	
pMGchbD	pMG36e with a fragment carrying a 296-bp deletion at the *chbD*-coding region and expressing a sgRNA specific of *chbD*	This work	
pRVchbC	pRV300 with a 639-bp *chbC* fragment	This work	
pRVchbR	pRV300 with a fragment carrying a 112-bp deletion at the *chbR*-coding region	This work	
pRVnagR	pRV300 with a frameshift at HindIII site in *nagR* fragment	This work	
pQE80	*E. coli* expression vector; Amp^R^	Qiagen	
pQEchbR	pQE80 containing *chbR*-coding region	This work	
pQEnagR	pQE80 containing *nagR*-coding region	This work	
pT1NX	Contains the P1 constitutive promoter, Erm^R^	(Schotte et al. 2000)	
pT1NchbD	pT1NX containing *chbD*-coding region	This work	

*CECT* Colección Española de Cultivos Tipo, *Erm*^*R*^ erythromycin resistant, *Amp*^*R*^ ampicillin resistant

The pRV300 (Leloup et al. 1997) and pMG36e (Gu et al. 2022) vectors were used for cloning experiments with *E. coli*, as well as for gene insertion and inactivation in *L. paracasei*. The pQE80 vector (Qiagen) was used for protein overproduction. The *E. coli*, *L. casei*, and *L. lactis* strains were transformed by electroporation using a Gene Pulser apparatus (Bio-Rad Laboratories), according to the manufacturer’s recommendations for *E. coli* and the methods described earlier for *L. casei* (Posno et al. 1991) and for *L. lactis* (Holo and Nes 1989).

### Culture of *L. paracasei* strains with monosaccharides and oligosaccharides

*L. paracasei* strains were cultured overnight at 37 °C under static conditions in sugar-free MRS basal medium. This medium contained bactopeptone (Gibco), 10 g L^−1^; yeast extract (Life Technologies), 4 g L^−1^; sodium acetate, 5 g L^−1^; triammonium citrate, 2 g L^−1^; magnesium sulfate 7-hydrate, 0.2 g L^−1^; manganese sulfate monohydrate, 0.05 g L^−1^; and Tween 80, 1 mL L^−1^. Overnight cultures were diluted to an OD_550_ of 0.05 in 100 µL of MRS basal medium containing 4 mM of *N*,*N*′-diacetylchitobiose (ChbNAc), *N*,*N′*,*N″*-triacetylchitotriose (TriChbNAc), *N*,*N′*,*N″,N′″*-tetracetylchitotriose (TetraChbNAc), chitobiose (Chb), *N*-acetylglucosamine (GlcNAc), or glucose. ChbNAc, triChbNAc, tetraChbNAc, Chb, and GlcNAc were obtained from Biosynth Ltd. (Compton, Berskhire, UK). Bacterial growth was monitored for 24 h by spectrophotometric measurements every 30 min at 550 nm in 96-well plates at 37 °C without shaking in an SPECTROstar Nano microplate reader (BMG Labtech, Ortenberg, Germany). At least three independent biological replicates were obtained for each growth curve. Results were expressed as means ± standard deviations.

### Construction of *L. paracasei* mutants in *chb* and *nag* genes

*L. paracasei* BL23 chromosomal DNA was isolated using the previously described protocol (Posno et al. 1991) and used as template in PCR reactions performed with Phusion High-Fidelity Polimerasa (Thermo Fisher Scientific) to obtain DNA fragments of *chb* and *nag* genes. The primer pairs used to amplify the *chbC*, *chbE*, *chbR*, and *nagR* fragments were 30200For/30200Rev, Hipo2For/Hipo2Rev, chb2BFor/chbR2Rev, and NagASalI/NagRrev, respectively (Table S1). The *chbC*, *chbE*, *chbR* and *nagR* fragments were cloned into pRV300 digested with EcoRV. The resulting plasmid containing the *chbC* fragment, pRVchbC, was used to transform the BL23 strain and single cross-over integrants were selected by resistance to erythromycin, confirmed by PCR analysis and DNA sequencing, which was performed by Eurofins Genomics (http://www.eurofinsgenomics.com). One mutant was selected and named BL402 (Table 1). The resulting plasmids containing the *chbE* and *nagR* fragments were digested at the unique NcoI and HindIII restriction sites to introduce a frameshift into the *chbE* and *nagR* coding regions, respectively. The digested plasmids were then treated with the Klenow fragment of the DNA polymerase I, ligated and transformed into *E. coli* DH10B. The resulting plasmids pRVchbE and pRVnagR were transformed into *L. paracasei* BL23 and erythromycin-resistant clones were obtained by plasmid integration. Using a double recombination strategy (Bidart et al. 2014), antibiotic-sensitive clones were isolated. One clone for each gene was selected that carried *chbE* and *nagR* mutations and was named V02 and BL404, respectively (Table 1). The resulting plasmid containing the *chbR* fragment was used as a template in a PCR reaction with the primers ChbRvFor and ChbRvRev (Table S1) to introduce a 112-bp deletion and frameshift in the *chbR* coding region. The resulting plasmid, pRVchbR, was transformed into *L. paracasei* BL23 and after applying a double recombination strategy, one clone that carried the *chbR* mutation was selected and named V03 (Table 1). To construct a *chbD* mutant, the endogenous CRISPR-Cas9 system of *L. paracasei* BL23 was used as previously described (Gu et al. 2022). Two *chbD* fragments were obtained in PCR reactions using the primer pairs Oligo-1F/Oligo-1R and Oligo-2F/Oligo-2R to introduce a 296-bp in-frame deletion in the *chbD* coding region. A 291-bp sequence encoding the sgRNA expression module (Gu et al. 2022) containing a 30-bp (5′-CTGGCAGTCTCAATGGTTTCTCTTACAAGA) sequence targeting the *chbD* gene, adjacent to a PAM sequence (5′-AGAAA) and located in the 296-bp deleted region, was synthetized by GeneArt (Thermo Fisher Scientific). This 291-bp sequence was amplified by PCR with primers Oligo_srF and F2-Rev. The three PCR fragments were cloned into pMG36e using the GeneArt™ Gibson Assembly® EX Cloning Kit (Thermo Fisher Scientific). The resulting plasmid, pMGchbD, was used to transform *L. paracasei* BL23, and clones were selected by resistance to erythromycin. In these clones, the presence of the *chbD* deletion was confirmed by PCR analysis (more than 90% positive clones) followed by DNA sequencing. One clone was selected and cultured without erythromycin for several generations for curing the pMGchbD plasmid. One antibiotic-sensitive clone was selected and named V19 (Table 1).

### Complementation of the *chbD* mutant

The *chbD* coding region was amplified by PCR using *L. paracasei* BL23 chromosomal DNA as a template and primers CD-F and CD-R (Table S1). The PCR fragment was cloned into pT1NX, which had previously been amplified in a PCR reaction using primers pT1NX-F and pT1NX-R, with the GeneArt™ Gibson Assembly® EX Cloning Kit (Thermo Fisher Scientific). The resulting plasmid, pT1NchbD, was then used to transform the *L. paracasei chbD* mutant V19. One clone was selected and named V37. Strain V19 was also transformed with the empty pT1NX vector to be used as a control, and one transformant was selected and named V36 (Table 1).

### Carbohydrate analysis

To determine the carbohydrates present in the supernatants from the *L. paracasei* cultures, the cells were removed by centrifugation and the cultures were analyzed by high-pH anion-exchange chromatography with pulsed amperometric detection (HPAEC-PAD) in a Dionex ICS3000 system using a CarboPac™PA100 analytical column equipped with a CarboPac™PA100 guard column (Dionex Corp., Sunnyvale, CA, USA). A gradient of 10 to 100 mM NaOH in 15 min with a flow rate of 1 mL min^−1^ was used at 27 °C. Monosaccharides and oligosaccharides were confirmed by comparison of their retention times with those of standards.

### RNA isolation and reverse transcription-quantitative PCR (RT-qPCR)

Total RNA was isolated from *L. paracasei* strains cultured in MRS basal medium containing 4 mM of different sugars as previously described (Bidart et al. 2014). The RNA was digested with DNaseI (Thermo Fisher Scientific) and retrotranscribed using the Maxima First-Strand cDNA Synthesis Kit for RT-qPCR (Thermo Fisher Scientific) (Bidart et al. 2014). RT-qPCR was performed for each cDNA sample in triplicate using the Lightcycler 480 (Roche), NZYSpeedy qPCR Green Master Mix (2X) (NZYtech) and the primer pairs: qChbAfor/qChb2Arev (*chbA*), qChb2Bfor/qChb2Brev (*chbB*), qChb2Cfor/qChb2Crev (*chbC*), qChbRfor/qChbRrev (*chbR*), qChbhipo1for/qChbhipo1rev (*chbD*), qChbhipo2for/qChbhipo2rev (*chbE*), qNagAfor/qNagArev (*nagA*), qNagRfor/qNagRrev (*nagR*), qNagBfor/qNagBrev (*nagB*) (Table S1). The reaction mixture (10 µL) contained 5 µL of 2X master mix, 0.5 µL of each primer (10 µM), and 2 µL of a 5–tenfold diluted sample of the cDNA synthesis reaction. Control reaction mixtures and cycle conditions were as previously described (Bidart et al. 2014). For each primer set, the cycle threshold values [crossover point (CP)] were determined using the automated method implemented in LightCycler 480 1.5.0 software (Roche). The *pyrG*, *ileS*, and *lepA* genes were selected as reference genes based on previous studies (Landete et al. 2010). Relative expression based on the expression ratio between target genes and reference genes was calculated using the software tool REST9000 (Pfaffl et al. 2002). Linearity and amplification efficiency were determined for each pair of primers. Each RT-qPCR was performed at least in triplicate with cDNA from two independent biological samples.

### Expression and purification of His-tagged ChbR and NagR

The coding regions of *chbR* and *nagR* were amplified by PCR using chromosomal DNA of *L. paracasei* BL23 as a template and the primer pairs: chbRB-Hfor/chbRB-Hrev; nagRP-Hfor/nagRP-Hrev, respectively. PCR fragments were cloned in pQE80 using the GeneArt™ Gibson Assembly® EX Cloning Kit (Thermo Fisher Scientific). The resulting plasmids pQEchbR and pQEnagR were used to transform *E. coli* NZY5α. The inserted sequence was confirmed by DNA sequencing, and one clone with the correct sequence was cultured overnight in 250 mL of Luria–Bertani medium (Oxoid) at 37 °C with agitation. When the culture reached an OD_595_ of 0.7, isopropyl-β-D-1-thiogalactopyranoside (IPTG) was added to 0.2 mM for protein induction, and incubation was continued at 18 °C for 18 h. Cells were harvested by centrifugation and resuspended in lysis buffer (phosphate buffered saline (PBS); lysozyme, 1 mg mL^−1^; dithiothreitol, 1 mM; phenylmethylsulphonyl fluoride, 1 mM) for 30 min at room temperature. Cell extracts were prepared, loaded onto a Ni Sepharose 6 Fast Flow column (GE Healthcare) and the proteins purified as previously described (Bidart et al. 2014). The fractions containing the proteins of interest were analyzed using SDS-PAGE gels, combined and dialyzed against PBS containing 20% glycerol. The purified proteins were kept frozen at −80 °C. Protein concentrations were determined with the Protein Assay Dye Reagent Concentrate (BioRad).

### Electrophoretic gel mobility shift assay (EMSA)

The upstream regions of the *chbC*, *chbR*, *nagA*, and *nagB* genes were amplified by PCR using chromosomal DNA from *L. paracasei* BL23 and the following primer combinations: EMSArcF/EMSArcR; EMSArbF/EMSArbR; EMSAaF/EMSAaR; and nagBF/nagBR, respectively. The first primer pair amplified a 160-bp DNA fragment from the *chbC* and *chbR* intergenic region; the second produced a 124-bp fragment from the *chbR* and *chbB* intergenic region. The third primer pair amplified a 118-bp DNA fragment upstream of the *nagA* gene and the last primer pair amplified a 183-bp DNA fragment upstream of the *nagB* gene. The different DNA fragments were used in electrophoretic mobility change assays with His-tagged ChbR or NagR. The binding assay was performed using different concentrations of either ChbR or NagR in a binding buffer containing Tris–HCl 25 mM, pH 7.5, MgCl_2_ 5 mM, DTT 1 mM, EDTA 1 mM, NaCl 100 mM and glycerol 10%, and with 0.25 µg of non-specific DNA (salmon sperm) and 0.20 µg of target DNA. Binding mixtures were incubated for 30 min at 37 °C and separated in 6% polyacrylamide gels in TAE buffer (40 mM Tris–HCl, pH 7.5, 20 mM acetic acid and 1 mM EDTA) at 60 V for 75 min. The DNA was stained with RedSafe™ (iNtRON). The images were captured using an Amersham imager 680 UV.

### Sequence analysis

The sequences of the *L. paracasei* BL23 *chb* and *nag* genes were retrieved from the GenBank database (accession no. FM177140). Gene and protein similarity searches were performed with BLAST (https://blast.ncbi.nlm.nih.gov/Blast.cgi). Transcriptional terminators and putative promoter sequences were analyzed by FindTerm and BPROM tools, respectively (http://www.softberry.com). Potential ChbR and NagR binding motifs were identified with MEME (https://meme-suite.org). Genomic context analysis was performed with the Graphics tool on genomes deposited at NCBI (https://www.ncbi.nlm.nih.gov/nuccore/).

## Results

### *Lacticaseibacillus paracasei* ferments *N,N′*-diacetylchitobiose (ChbNAc) and *N,N′,N″*-triacetylchitotriose (TriChbNAc) and transports them by using a cellobiose-class PTS

**Fig. 2:**
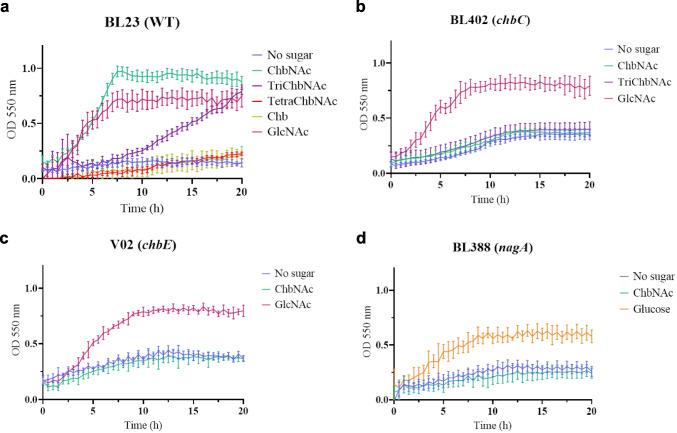
Growth curves of *Lacticaseibacillus paracasei* wild type and different mutant strains. **a** Wild type strain BL23 on MRS basal medium without carbon source (blue), with *N*,*N′*-diacetylchitobiose (ChbNAc) (green), *N*,*N′*,*N″*-triacetylchitotriose (TriChbNAc) (purple), *N*,*N′*,*N″,N′″*-tetracetylchitotriose (TetraChbNAc) (red), chitobiose (Chb) (yellow) or *N*-acetylglucosamine (GlcNAc) (magenta). **b** Mutant strain BL402 (*chbC*) on MRS basal medium without carbon source (blue), with ChbNAc (green), TriChbNAc (purple) or GlcNAc (magenta). **c** Mutant strain V02 (*chbE*) on MRS basal medium without carbon source (blue), with *N*,*N′*-diacetylchitobiose (ChbNAc) (green) or *N*-acetylglucosamine (GlcNAc) (magenta). **d** Mutant strain V06 (*nagA*) on MRS basal medium without carbon source (blue), with *N*,*N′*-diacetylchitobiose (ChbNAc) (green) or glucose (orange). Data presented are mean values based on at least three replicates. Error bars indicate standard deviations

Previous results from our laboratory showed that *L. paracasei* BL23 cannot use fucosyl-α−1,6-*N*,*N*′-diacetylchitobiose (N2F *N*-glycan), the core fucosylated disaccharide of *N*-glycosylation, unless the gene encoding the AlfR2 repressor of the *alf-2* operon had been inactivated. In this case, the N2F *N*-glycan was transported due to constitutive expression of the AlfH permease (Becerra et al. 2020). Since, contrarily to N2F *N*-glycan, most *N*-linked glycans are not fucosylated, we tested whether *L. paracasei* was able to metabolize the unfucosylated core disaccharide *N*,*N*′-diacetylchitobiose (ChbNAc). Growth of *L. paracasei* BL23 using MRS basal medium supplemented with 4 mM ChbNAc showed that this strain was able to catabolize this disaccharide (Fig. 2a). The growth patterns of *L. paracasei* in non-supplemented MRS basal medium and supplemented with *N*-acetylglucosamine (GlcNAc) as negative and positive controls, respectively, are also shown (Fig. 2a). We next tested whether, similar to N2F *N*-glycan, the AlfH permease was involved in the uptake of ChbNAc. The results showed that a mutant in *alfH* (strain BL372) (Becerra et al. 2020) had a similar growth pattern to the wild-type strain, indicating that the AlfH permease was not involved in the transport of ChbNAc (Supplementary Fig. S1).

In order to identify the permease required for the transport of ChbNAc in *L. paracasei*, the sequence of the IIC protein from the PTS involved in the uptake of this disaccharide in *Bacillus cereus* (Cao et al. 2011) was used in a BLAST search against the *L. paracasei* BL23 genome (GenBank accession no. FM177140) (Maze et al. 2010). The BLAST hit with maximum sequence identity (33%) corresponded to the product of the gene *pts7C* (LCABL_ RS14765), annotated as a IIC subunit of a cellobiose-class PTS. Analysis of the DNA region around this gene, renamed here as *chbC*, showed the presence of two genes transcribed in the opposite direction (*chbBA*; LCABL_RS14775 and LCABL_ RS14780, respectively), which also encoded IIB and IIA domains of a cellobiose-class PTS (Fig. 1a). To determine whether this PTS was involved in the internalization of ChbNAc, a mutant disrupted in *chbC* (strain BL402) was constructed. This strain lost its capacity to grow with ChbNAc as a carbon source (Fig. 2b). Analysis of sugar content in the culture supernatants showed that the disaccharide was not consumed, demonstrating that the IIC domain encoded by *chbC* was involved in ChbNAc utilization and suggesting that *chbC* and *chbBA* genes encoded a PTS^Chb^ for the uptake of ChbNAc in *L. paracasei* BL23 (Supplementary Fig. S2).

ChbNAc is also a degradation product of the abundant polysaccharide chitin. Therefore, we tested if *L. paracasei* could be cultured in the presence of the larger chitin oligosaccharides *N*,*N′*,*N″*-triacetylchitotriose (TriChbNAc) and *N*,*N′*,*N″,N′″*-tetracetylchitotriose (TetraChbNAc). *L. paracasei* BL23 was able to metabolize the tri-saccharide but not the tetra-saccharide (Fig. 2a). We next tested the involvement of the identified PTS^Chb^ in the transport of TriChbNAc. The BL402 (*chbC*) strain did not grow in the presence of this trisaccharide, indicating that its utilization was also dependent on PTS^Chb^ (Fig. 2b). The deacetylated disaccharide chitobiose (glucosamine-β−1,4-glucosamine) was not utilized by *L. paracasei* BL23 (Fig. 2a).

### The *chbE* and *chbD* genes are involved in the metabolism of ChbNAc

The *chbC* gene was followed by two other genes (*chbD* and *chbE*) of unidentified function that were transcribed in the same direction, probably forming an operon (Fig. 1a). A BLAST search with the deduced amino acid sequence of ChbE evidenced 26% identities and 43% positive sequence identity to the MupG family TIM beta-alpha barrel fold protein of *Staphylococcus aureus* (Kluj et al. 2018). This protein has demonstrated 6-phospho-*N*-acetylmuraminidase activity, hydrolyzing the phosphorylated disaccharide *N*-acetylmuramic acid-β−1,4-GlcNAc into *N*-acetylmuramic-6P and GlcNAc, and it was recently employed to describe the new GH170 family of glycosyl hydrolases. To determine whether the hypothetical glycosyl hydrolase ChbE was involved in the metabolism of ChbNAc in *L. paracasei* BL23, a mutant in *chbE* (strain V02) was constructed. This mutant showed a diminished growth with ChbNAc which was similar to that of the growth in the non-supplemented MRS basal medium used as a negative control (Fig. 2c), indicating that ChbE was necessary for the utilization of this carbohydrate.

**Fig. 3:**
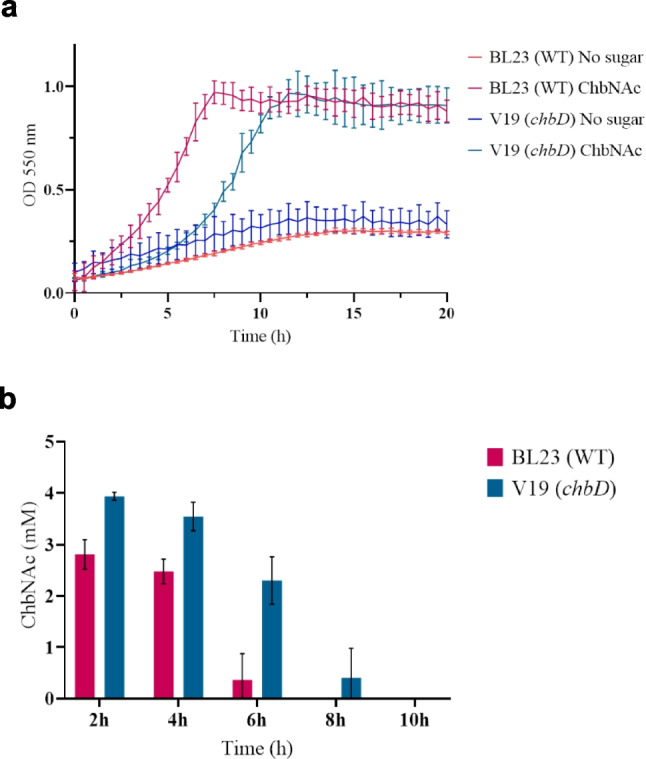
Effect of *chbD* on *N*,*N′*-diacetylchitobiose (ChbNAc) metabolism in *Lacticaseibacillus paracasei*. **a** Growth curves of *L. paracasei* mutant strain V19 (*chbD*) on MRS basal medium without carbon source (blue) and with 4 mM ChbNAc (teal). The growth pattern of wild-type (WT) strain BL23 is presented for a better comparison. Data presented are mean values based on at least three replicates. Error bars indicate standard deviations. **b** Residual ChbNAc present in the supernatants along the growth curves at 2, 4, 6, 8 and 10 h cultures of *L. paracasei* strains BL23 (WT) (magenta) and V19 (*chbD*) (teal) on MRS basal medium supplemented with 4 mM ChbNAc. Data presented are mean values based on at least two replicates. Error bars indicate standard deviations

The product of *chbD* was a 139 amino acids protein containing the domain of unknown function DUF3284. A mutant with an in-frame deletion of *chbD* (strain V19) showed a longer lag phase when cultured on ChbNAc compared to the wild-type. However, the maximum cell density reached was similar for both strains (Fig. 3a). This was consistent with the residual ChbNAc present in the supernatants along the growth curves. At 2 and 4 h of culture, the amount of disaccharide not consumed was higher in the mutant than in the wild-type, and at 6 h about half of the amount of disaccharide remained in the supernatant of the *chbD* mutant, while it was almost depleted in the wild-type supernatant (Fig. 3b). To determine if the longer lag phase was due to the absence of ChbD and not to a polar effect on the downstream *chbE* gene, V19 was transformed with pT1NchbD (Table 1), which constitutively expresses *chbD* in *trans*. The results showed that the growth rate was partially restored in the complemented strain (V37). Curiously, the *chbD* mutant transformed with the empty vector pT1NX showed a diminished growth rate, suggesting that the presence of the replicating plasmid increased the growth impairment of the *chbD* mutant when cultured with ChbNAc (Supplementary Fig. S3).

### The *nagA* gene is involved in the metabolism of ChbNAc

The above results indicated that *chbC* and *chbE* are required for ChbNAc utilization in *L. paracasei* BL23. Therefore, ChbNAc-P resulting from the transport via the PTS^Chb^ and the activity of the ChbE, a likely phospho-β-*N*-acetylglucosaminidase, would result in the metabolites GlcNAc-6P and GlcNAc (Fig. 1b). To analyze whether the catabolism of the generated GlcNAc-6P was dependent on the *N*-acetylglucosamine-6P deacetylase encoded by the gene *nagA* present in the *L. paracasei* BL23 genome (LCABL_RS09900) (Fig. 1a), a previously constructed *nagA* mutant (strain BL388) (Bidart et al. 2014) was cultured on ChbNAc, confirming that it was unable to grow with this disaccharide (Fig. 2d). In agreement with previous results that showed that BL388 cannot utilize GlcNAc (Bidart et al. 2014), this indicated that *nagA* was involved in the metabolism of GlcNAc derived from ChbNAc.

*L. paracasei* BL23 also carried a second *nag* gene putatively involved in GlcNAc metabolism (*nagB;* LCABL_RS15180), which encodes a glucosamine-6P deaminase required for the entry of the glucosamine-6P derived from NagA activity into the glycolysis. Several attempts to construct a *nagB* mutant in *L. paracasei* were unsuccessful (data not shown), suggesting that this activity may be essential.

### Transcriptional analyses of the *chb* and *nag* genes

**Fig. 4:**
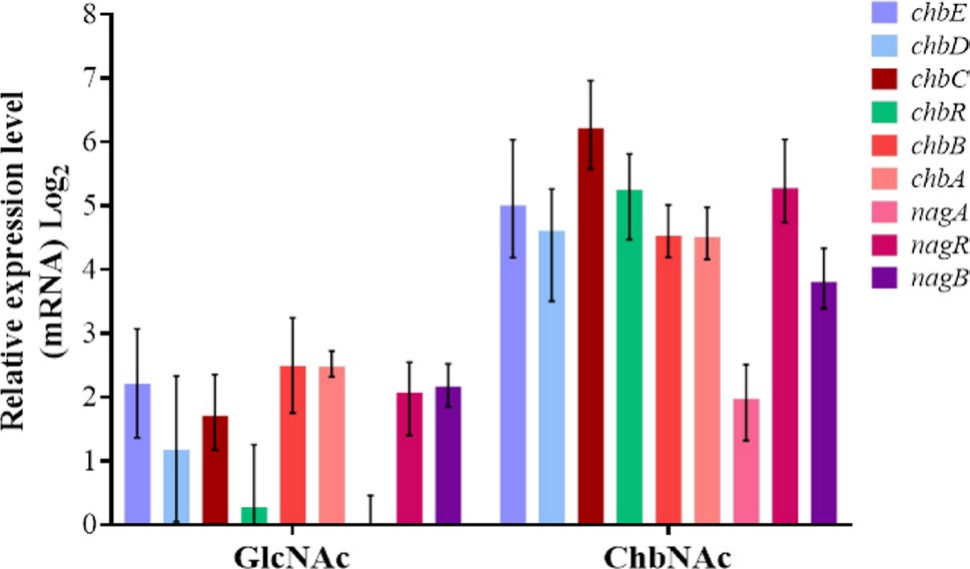
Transcription analysis by RT-qPCR of the expression of *chb* and *nag* genes in *Lacticaseibacillus paracasei* BL23 grown in MRS basal medium containing *N*-acetylglucosamine (GlcNAc) or *N*,*N*′-diacetylchitobiose (ChbNAc). Cells grown in MRS basal medium with glucose were used as reference condition. Data presented are mean values based on three replicates of at least two biological independent samples. Bars indicate standards errors

Gene expression analysis of the *chb* and *nag* genes was performed with RNA isolated from *L. paracasei* BL23 cells grown in MRS basal medium containing ChbNAc, GlcNAc, and glucose (Fig. 4). Taking as a reference the transcript levels in cells growing with glucose, all the genes tested were induced by ChbNAc. The induction of the *chb* and *nag* genes ranged from 22.7- to 74.0-fold and from 3.9- to 38.7-fold, respectively. In contrast, not all the *chb* or *nag* genes were induced in the presence of GlcNAc, the hexose constituent of ChbNAc, and those that were induced showed lower levels than that observed with ChbNAc. Remarkably, the qPCR analysis showed no induction of *nagA* by GlcNAc.

**Fig. 5:**
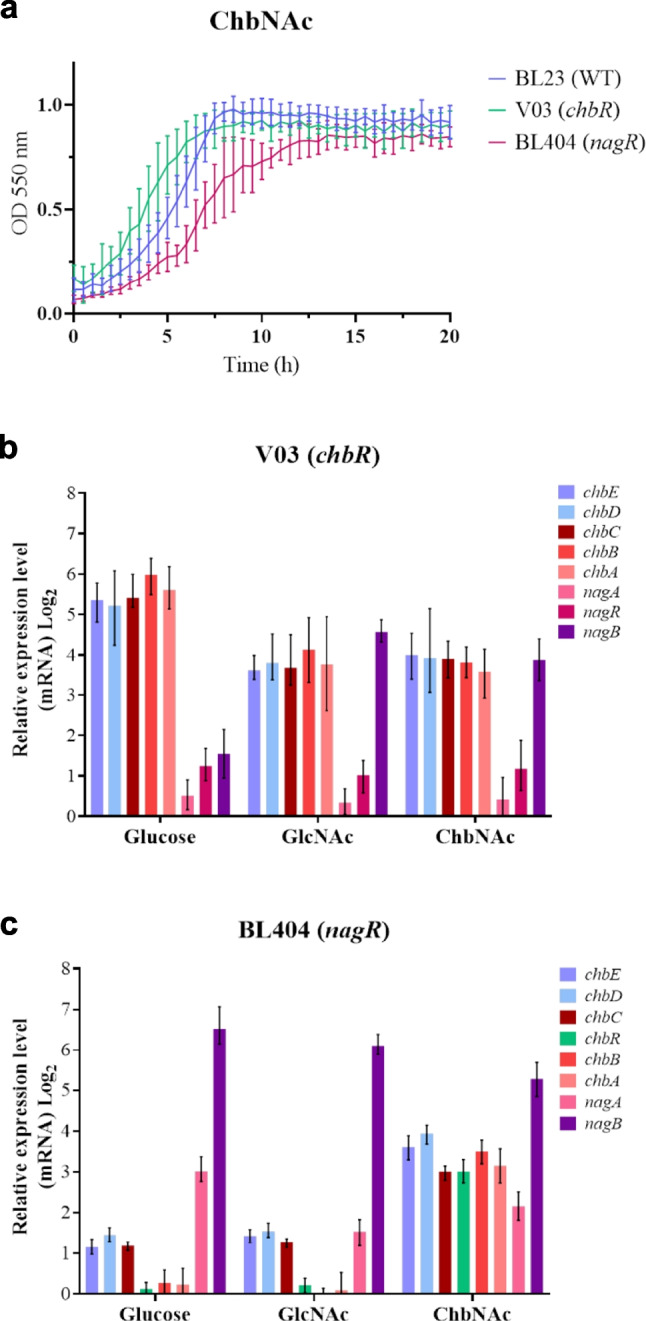
Effect of mutations in transcriptional regulators on growth and gene expression. **a** Growth curves of *Lacticaseibacillus paracasei* BL23 (blue), mutant strains V03 (*chbR*) (green) and BL404 (*nagR*) (magenta) on MRS basal medium with *N*,*N′*-diacetylchitobiose (ChbNAc). Transcription analysis by RT-qPCR of the expression of *chb* and *nag* genes in *L. paracasei* mutant strains V03 (*chbR*) (**b**) and BL404 (*nagR*) (**c**) grown in MRS basal medium containing glucose, *N*-acetylglucosamine (GlcNAc) or *N*,*N*′-diacetylchitobiose (ChbNAc). *L. paracasei* BL23 grown in MRS basal medium with glucose was used as reference strain. Data presented are mean values based on three replicates of at least two biological independent samples. Bars indicate standards errors

Upstream of *chbC,* and transcribed in the same direction, a gene (*chbR*; LCABL_RS14770) was present encoding a putative transcriptional regulator of the GntR family. Similarly, *nagA* formed an operon structure with *nagR* (LCABL_RS09895), which also encoded a GntR-class regulator. Both proteins contained a winged helix-turn-helix (WHTH) DNA-binding domain (amino acid residues 2 to 62 and 4 to 69, respectively). To analyze the involvement of these putative regulators in the control of ChbNAc metabolism in *L. paracasei* BL23, mutants disrupted in *chbR* (strain V03) and *nagR* (strain BL404) were constructed. In MRS basal medium supplemented with ChbNAc, these mutants did not display growth impairment, although the *nagR* strain grew slower compared to the wild type (Fig. 5a). Compared to glucose-grown wild-type cells, the expression of *chb* genes in the *chbR* strain was higher with this non-inducing sugar and at a similar level as that found after growth with GlcNAc or ChbNAc. This indicated that ChbR acted as a transcriptional repressor of the *chb* genes (Fig. 5b). In the *chbR* mutant strain, transcription of *nagB* was induced by growth in GlcNAc. Notably, the fold-induction observed in this strain was higher than that in the wild type (4.4-fold in wild type vs. 23.7-fold in *chbR*), suggesting that ChbR may exert a repressive effect on *nagB* expression under these conditions (Fig. 5b).

Unlike ChbR, the transcriptional regulator NagR did not appear to influence the expression of *chb* genes, as their transcription levels in the BL404 (*nagR*) mutant strain remained largely unchanged in both glucose and GlcNAc compared to the wild-type strain (Fig. 5c). In contrast, *nagA*, and specially *nagB*, were clearly repressed by NagR, as their expression levels were consistently higher in the *nagR* mutant across all tested sugars. (Fig. 5c).

### ChbR binds the *chb* promoter, NagR binds *nagAR*, and both bind *nagB*.

**Fig. 6:**
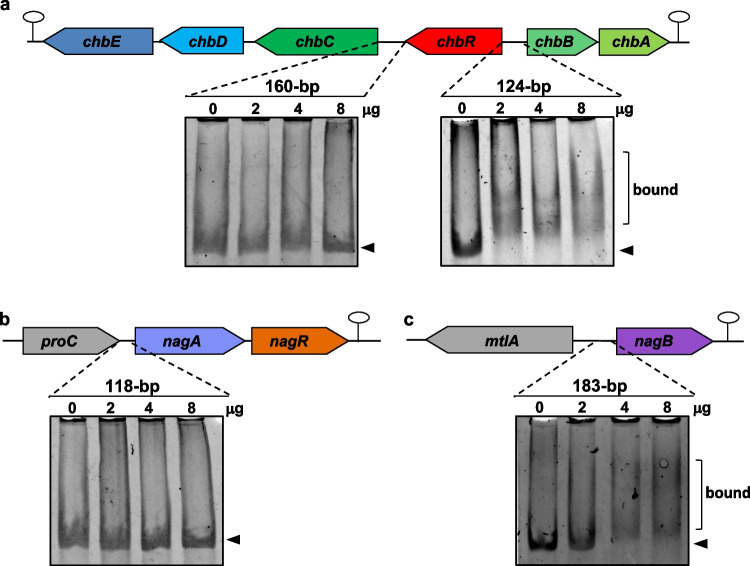
Binding of ChbR to different promoters. Electrophoretic gel mobility shift assay (EMSA) with the 160-bp and 124-bp DNA fragments of the intergenic region between *chbC* and *chbR*, and *chbR* and *chbB* genes, respectively (**a**), with the 118-bp and 183-bp DNA fragments of the intergenic region between *proC* and *nagA* genes (**b**), and *mtlA* and *nagB* genes (**c**), with 0 µg, 2 µg, 4 µg, or 8 µg of His-tagged ChbR

**Fig. 7:**
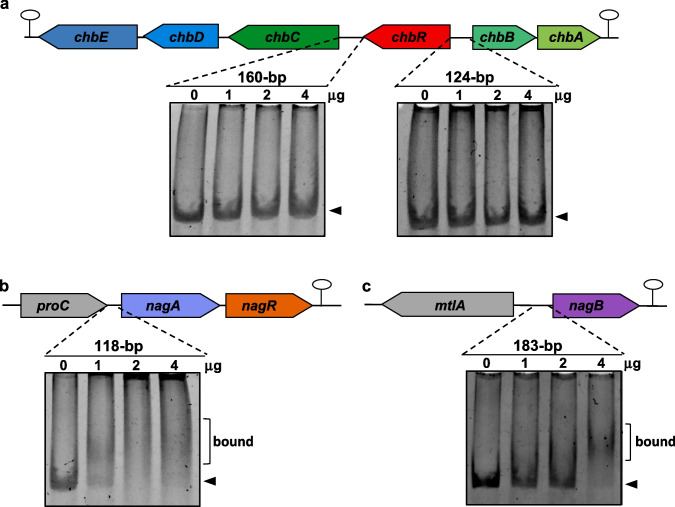
Binding of NagR to different promoters. Electrophoretic gel mobility shift assay (EMSA) with the 160-bp and 124-bp DNA fragments of the intergenic region between *chbC* and *chbR*, and *chbR* and *chbB* genes, respectively (**a**), with the 118-bp and 183-bp DNA fragments of the intergenic region between *proC* and *nagA* genes (**b**), and *mtlA* and *nagB* genes (**c**), with 0 µg, 1 µg, 2 µg, or 4 µg of His-tagged NagR

To analyze whether the regulatory effect of ChbR and NagR required a direct interaction with the promoter regions of the *chb* and *nag* genes, ChbR and NagR were purified as His-tagged proteins. These proteins were used in EMSA assays with four different DNA fragments covering the intergenic regions between *chbC* and *ChbR*, *chbR*, and *chbB*, and the *nagA* and *nagB* promoter regions, respectively. The results showed that ChbR retarded the *chbR-chbB* intergenic fragment, but not the *chbC-chbR* fragment (Fig. 6). This identified the regions upstream of *chbR* and *chbB* as targets for regulation and likely containing the promoters for the *chbRCDE* and *chbBA* units, respectively. In addition, a weak interaction of ChbR was evidenced with the *nagB* promoter, but not with the *nagA* promoter (Fig. 6). NagR bound both the *nagA* and *nagB* promoter regions, but did not interact with the *chb* fragments (Fig. 7). These data were consistent with the results showing that ChbR acted as a transcriptional repressor of the *chb* cluster and that NagR regulated the *nagAR* cluster. In addition, they were in agreement with qPCR experiments showing that *nagB* was regulated by ChbR as well as NagR. In silico analysis of the DNA sequences to which the transcriptional regulators ChbR and NagR bound revealed putative binding sites consisting of in partially palindromic 14-bp sequences (Supplementary Fig. S4). The identified motifs from the ChbR- and NagR-bound fragments were coincident, with bases fully conserved at positions 2, 7, 8 and 13. They were consistent with the DNA-binding sequences previously characterized for other NagR orthologs; notably in *B. subtilis*, where NagR binds to the promoter regions of *nagA* and *nagP*, the latter encoding a PTS EIIBC component for GlcNAc (Bertram et al. 2011). Similar binding sites have also been identified for DasR, a regulator of GlcNAc and chitin utilization genes in *S. coelicolor* (Colson et al. 2007; Rigali et al. 2006). These sites typically span 16 bp and exhibit conserved sequence motifs, which were also present in the putative NagR binding sites described here (Supplementary Fig. S5). These potential binding motifs partially overlapped the predicted −10 boxes of the promoter regions, which agreed with their function as repressors (Supplementary Fig. S4). Remarkably, despite the substantial conservation of their binding sites, ChbR did not interact in vitro with the *nagAR* promoter, and conversely, NagR did not bind to the *chb* promoter.

### Genetic organization of presumed *chb* operons in other bacteria

**Fig. 8:**
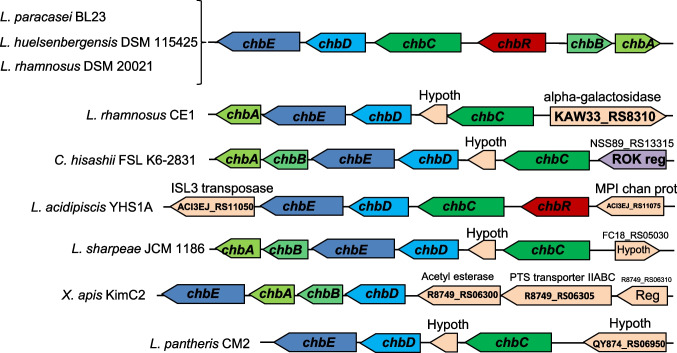
Structural organization of putative *chb* gene clusters in representative microorganisms: *L. huelsenbergensis* (*Lacticaseibacillus huelsenbergensis*), *L. rhamnosus* (*Lacticaseibacillus rhamnosus*), *C. hisashii* (*Caldifermentibacillus hisashii*), *L. acidipiscis* (*Ligilactobacillus acidipiscis*), *L. sharpeae* (*Lacticaseibacillus sharpeae*), *X. apis* (*Xylocopilactobacillus apis*) and *L. pantheris* (*Lacticaseibacillus pantheris)*. The organization of the *L. paracasei* (*Lacticaseibacillus paracasei*) BL23 *chb* gene clusters is also shown for a better comparison. Hypoth, hypothetical protein; ROK reg, ROK family transcriptional regulator; MPI chan prot, MPI family channel protein; PTS, phosphoenolpyruvate-dependent sugar phosphotransferase system; Reg, transcriptional antiterminator BglG family

To analyze the presence of putative *chb* operons in other bacteria, a BLAST search was performed using the ChbD protein against the NCBI ClusteredNR database, as the *L. paracasei chb* operon is remarkable for encoding this protein. Sixty-one clusters of ChbD homologs were identified in additional strains belonging to several genera, including *Lacticaseibacillus*, *Lactiplantibacillus*, *Lactobacillus*, *Ligilactobacillus, Caldifermentibacillus* and *Xylocopilactobacillus*, with protein identities ranging from 30 to 100%. While not all *chbD* homologs clustered with *chbE* genes, they were usually found in close proximity to genes encoding PTS transporters. The genetic context of gene clusters encoding both ChbD and ChbE orthologs shows that the gene content and order present in the *L. paracasei chb* clusters are conserved in certain *Lacticaseibacillus huelsenbergensis* and *Lacticaseibacillus rhamnosus* strains (Fig. 8). Nevertheless, other strains of *L. rhamnosus* are devoid of a complete PTS^chb^, as they lack the IIB domain of the PTS and also lack the transcriptional regulator-encoding genes (Fig. 8). This latest organization is also found in some *L. paracasei* and *Lacticaseibacillus casei* strains (https://www.ncbi.nlm.nih.gov/nuccore/). *Caldifermentibacillus hisashii* contains a whole *chb* cluster with a different gene order and a different transcriptional regulator belonging to the ROK family. Other species that contain homologs of *chbD* and *chbE* either lack the gene that encodes the transcriptional regulator; lack some of the genes that encode the PTS proteins, or both (Fig. 8). Whether those clusters have a role in ChbNAc metabolism in the bacterial strains that harbor them remains to be elucidated.

## Discussion

*N*,*N*′-Diacetylchitobiose (ChbNAc) is a component of *N*-glycosylated proteins and is also the major chitin degradation product by the action of chitinases (Ren et al. 2022; Yamaguchi et al. 2024). Most studies on the catabolism of this disaccharide in bacteria have focused on the genera *Vibrio*, *Serratia*, *Bacillus, Paenibacillus*, and *Streptomyces*, whose species play an important role in chitin recycling in marine and soil environments (Cao et al. 2011; Iinuma et al. 2018; Itoh et al. 2024; Kitaoku et al. 2021; Toratani et al. 2008). However, with the exception of *E. coli* (Walter et al. 2021), there is limited knowledge regarding the catabolism of ChbNAc in bacteria adapted to the human gastrointestinal tract. *L. paracasei*, a widely used probiotic species, is able to survive and transiently colonize the gut (Hill et al. 2018; Xiao et al. 2024). Therefore, the presence of genes involved in the utilization of dietary and host-derived glycans confers a competitive advantage for persistence in the gut. We have demonstrated here that *L. paracasei* BL23 is able to utilize ChbNAc as a source of carbon and energy for growth. The PTS transporter ChbABC and the glycosyl hydrolase ChbE, encoded by the divergently transcribed gene clusters *chbRCDE* and *chbBA*, are necessary for the metabolism of the disaccharide. In addition, the gene *nagA*, which encodes a GlcNAc-6P deacetylase, is also involved in the utilization of ChbNAc by *L. paracasei*. This agrees with a model where ChbNAc-P is produced by the action of PTS^chb^ and hydrolyzed by ChbE, producing GlcNAc-6P and GlcNAc. GlcNAc-6P can be further metabolized through the activities of NagA and NagB, but the metabolism of GlcNAc generated after ChbNAc-P hydrolysis requires phosphorylation by an as yet unknown intracellular kinase before it can be channeled through glycolysis. In *L. paracasei*, GlcNAc is a common intermediate in the catabolism of human milk and mucosa-derived glycans such as lacto-*N*-biose (Bidart et al. 2014), *N*-acetyllactosamine (Bidart et al. 2018), fucosyl-α−1,3-GlcNAc (Rodriguez-Diaz et al. 2012) and the glycoamino acid fucosyl-α−1,6-*N*-GlcNAc-Asn (Becerra et al. 2020). During the metabolism of this glycoamino acid in *L. paracasei*, the intracellularly liberated GlcNAc is phosphorylated by the SugK kinase, which is encoded in the inducible *alf-2* operon (Becerra et al. 2020). However, as occurs in the *chb* operon studied here, no sugar kinases are encoded in the operons involved in the metabolism of the other three aforementioned glycans (Bidart et al. 2014, 2018; Rodriguez-Diaz et al. 2012).

ChbE belongs to the recently described GH170 family (www.cazy.es). Among all glycosyl hydrolases that constitute this new family, only MupG from *S. aureus*, has been biochemically characterized as a 6-phospho-*N*-acetylmuramidase that participates in peptidoglycan recycling (Kluj et al. 2018). Our genetic data also point to ChbE as a 6-phosphoglycosyl hydrolase. Contrarily to that, in *E. coli* the phosphoglycosyl hydrolase associated with ChbNAc catabolism is a GH4 family enzyme that cleaves glucosamine-6P-glycosides. In this case, ChbNAc-P needs to be first deacetylated by the monodeacetylase ChbG encoded in the *E. coli chb* operon (Walter et al. 2021), before being a substrate for the GH4 enzyme. Other glycosidases that have been involved in the degradation of ChbNAc belong to different GH families. A GH20 enzyme is secreted to the medium and hydrolyzes ChbNAc extracellularly in *Serratia marcescens* (Vaaje-Kolstad et al. 2013), whereas GH3 enzymes act intracellularly on non-phosphorylated ChbNAc after being taken up by ABC transporters in *S. coelicolor* and *Paenibacillus* sp. (Itoh et al. 2019; Saito et al. 2013).

ChbD is classified as a member of Pfam11687 and contains the DUF3284 domain of unknown function. The *chbD* deletion mutant constructed here in *L. paracasei* BL23 exhibited an extended lag phase when grown on ChbNAc compared to the wild-type strain. This phenotype suggests a potential role for ChbD in optimizing the utilization of ChbNAc, although its precise function remains unknown. Similarly, the gene cluster involved in cellodextrins catabolism in *Enterococcus faecalis* encodes a protein homologous to ChbD (CelI), which is essential for cellotetraose utilization via a specific PTS (Combret et al. 2025). The DUF3284 family of proteins appears to be restricted to Bacillota (previously Firmicutes) (https://www.ncbi.nlm.nih.gov/Structure/cdd/) and although not all *chbD* homologs clustered with *chbE* genes, they were often found near genes that encode PTS transporters. Together with the observed phenotype of the *chbD* mutant and the inability of an *E. faecalis celI* mutant to utilize the PTS sugar cellotetraose (Combret et al. 2025), these findings suggest that proteins belonging to the DUF3284 family may play a previously unrecognized role in PTS-mediated transport. Although their exact function remains to be elucidated, these proteins could be involved in substrate recognition, stabilization of the transport complex, or coordination of downstream processing steps.

The results obtained in this work showed that the transcriptional regulation of the *chbRCDE* and *chbBA* gene clusters in *L. paracasei* involves induction by ChbNAc mediated by the transcriptional repressor ChbR. Based on our data, the phosphorylated product of ChbNAc transport via the PTS (ChbNAc-P) is likely the effector molecule for ChbR. This is supported by the observation that growth on GlcNAc, which should lead to the accumulation of common downstream metabolites such as GlcNAc-6P or glucosamine-6-P (GlcN-6P), did not induce *chb* gene expression. In addition, the *nagAR* and *nagB* genes, located at different chromosomal regions, are also induced by the growth of *L. paracasei* on ChbNAc, probably via these derived metabolites and the participation of NagR. In fact, analysis of the NagR ortholog in *B. subtilis* showed that GlcN-6P inhibited its DNA binding (Bertram et al. 2011). A similar situation was found for DasR binding to the *nagB* promoter in *S. coelicolor* (Rigali et al. 2006), suggesting that GlcN-6P may also be the effector of *L. paracasei* NagR. In this organism, this repressor acts on both *nag* clusters and, additionally, ChbR exerts also control on *nagB*, whereas NagR does not affect the transcription of *chb* genes. In *E. coli*, the *chb* operon is regulated by a complex network that includes its own encoded ChbR regulator, an AraC family regulator with repressor/activator dual function; NagC, a repressor of the ROK family encoded in the *nag* operon; and the cAMP/CAP complex (Plumbridge and Pellegrini 2004). *E. coli* ChbR and NagC act as repressors in the absence of ChbNAc and their concerted action ensures that the expression of the *chb* operon is tightly linked to that of the *nag* operon, which is necessary for the subsequent catabolism of GlcNAc derived from ChbNAc (Plumbridge and Pellegrini 2004). The observation that the *L. paracasei nagA* gene is repressed by NagR yet not induced by GlcNAc, despite being activated by ChbNAc, suggests a complex regulatory mechanism governing *nag* gene expression. This likely reflects the critical role of these genes in recycling GlcNAc derived from cell wall turnover (Reith and Mayer 2011). In *L. paracasei*, beyond the essential role of *nagA* in ChbNAc metabolism, the coordinated regulation of *chb* genes and *nagB* by ChbR also highlights the necessity for tightly integrated control between the ChbNAc and GlcNAc metabolic pathways.

This study reports the first characterization of the genes and catabolic pathway involved in ChbNAc utilization within the order *Lactobacillales*. The pathway exhibits distinctive features compared to those previously described in other bacterial groups. Although the associated PTS belongs to the cellobiose class, similar to that found in *E. coli*, the *L. paracasei chb* operon contains an accessory gene (*chbD*) of unknown function. In addition, the hydrolysis of the internalized and phosphorylated disaccharide is catalyzed by a glycosyl hydrolase from the relatively unexplored GH170 family. *L. paracasei* may use the *chb* and *nag* genes as part of a system for scavenging glycans derived from host mucosa and diet. Overall, this work provides new insights into how lactobacilli adapt to utilize specific glycans, potentially representing an evolutionary strategy for successful colonization of the gastrointestinal niche.

## Supplementary Information

Below is the link to the electronic supplementary material.

ESM1 (PDF 354 KB)

## Data Availability

All data generated or analyzed during this study are included in this published article and its supplementary information files.
