# Supplementary material for: Chb and nag genes drive N,N′-diacetylchitobiose metabolism in probiotic Lacticaseibacillus paracasei
Source: Appl Microbiol Biotechnol. 2026 Jan 10;110(1):7. doi: 10.1007/s00253-025-13656-2 (PMC12791066; doi:10.1007/s00253-025-13656-2)
Supplement: Supplementary file 1 — (PDF 354 KB) [file 253_2025_13656_MOESM1_ESM.pdf]

Supplementary Information

***Chb* and *nag* genes drive *N,N'-diacetylchitobiose* metabolism in probiotic *Lacticaseibacillus paracasei*.**

Víctor García-Telles[1] (ORCID: 0009-0007-4180-9083), Jimmy E. Becerra[1,a] (ORCID: 0000-0001-5499-0511), Jesús Rodríguez-Díaz[2,3] (ORCID: 0000-0002-9698-7684), Vicente Monedero[1#] (ORCID: 0000-0001-7461-8047) and María J. Yebra[1#] (ORCID: 0000-0003-4638-986X)

[1]Laboratorio de Bacterias Lácticas y Probióticos, Departamento de Biotecnología de Alimentos, Instituto de Agroquímica y Tecnología de Alimentos (IATA-CSIC), Valencia, Spain.

[2]Departamento de Microbiología, Facultad de Medicina, Universidad de Valencia, Valencia, Spain.

[3]INCLIVA. Instituto de Investigación Sanitaría del Hospital Clínico de Valencia. Valencia, Spain.

[a]Present address: Grupo de Investigación Alimentación y Comportamiento Humano, Universidad Metropolitana, Barranquilla, Colombia.

[#]Address correspondence to Vicente Monedero, vmon@iata.csic.es and María J. Yebra, yebra@iata.csic.es

Applied Microbiology and Biotechnology

TABLE S1. Primers used in this study

Name	Sequence
Hipo2For	5'-GGAATATCGCTCTATCCGG
Hipo2Rev	5'-TTTCAAGAACAAATGTTGACC
Chb2BFor	5'-ATGTTAATCACATCAACCGGA
ChbR2Rev	5'-GCTGACAGCGTCAGATTACGG
ChbRvFor	5'-GGACCGCTTGATATCGATAAGTCG
ChbRvRev	5'-GGGCAAAAATGATATCCAGCGCCT
30200For	5'-AGCAGCGATCAAGTCACTG
30200Rev	5'-ATCGATAATTTAGCAACCGC
NagASalI	5'-TTACCGTCGACATGCGTAGCAAAGGC
NagRrev	5'-GGCTTCTGTGCCTGTTCG
Oligo_2F	5'-TGTATGGCCAACTCATTCGATGGCAGCGTAAGCACCGGTT
Oligo_2R	5'-AAAGAAAGTTATGTGAGCTCGATATCGCCGCGTTTAAAGGA
Oligo_1F	5'-GAATCGCATCTGAAATATTCAGTAAGTTCTTTGGCGCATA
Oligo_1R	5'-AACCGGTGCTTACGCTGCCATCGAATGAGTTGGCCATACA
Oligo_srF	5'-TCCTTTAAACGCGGCGATATCGAGCTCACATAACTTTCTTT
F2-Rev	5'-TTCGTTTTCAGACTTTGCAATCTAGAAAAAAAATGTATGGCCCA
chbRB-Hfor	5'-CATCACCATCACCATCACGGATCCATGTATCACGATATTGCAGAG
chbRB-Hrev	5'-GTCCAAGCTCAGCTAATTAAGCTTTCATTGGTCCAAATCAATGTTG
nagRP-Hfor	5'-CATCACCATCACCATCACGGATCCATGGATGCACCAGTTTATATT
nagRP-Hrev	5'-GTCCAAGCTCAGCTAATTAAGCTTTTACCGTTCTAGATAAAATTC
qChb2Afor	5'-TTCACCAGCTAAATCGCGGA
qChb2Arev	5'-ATGTTAACCGCTGAAGCCCA
qChb2Bfor	5'-CCTTGAGGACTTTTTCGCCG
qChb2Brev	5'-GTTAGGACCTCAAGTCCGCT
qChb2Cfor	5'-TCGTGACGCTTGGTACCTTC
qChb2Crev	5'-TTGAAGAAGCCCCACATGCT
qChbRfor	5'-CAAACCGCGATGGTCAAAGG
qChbRrev	5'-TCCAAGTCGTACAACTGCCC
qChbhipo1for	5'-TGGGCTAATGGCATGACTGG
qChbhipo1rev	5'-GCGGTTTTGCCTTCAATCGT
qChbhipo2for	5'-TGGGGACTGCGACTAGATGA
qChbhipo2rev	5'-TCAGCCCGTATGGATTACGC
qNagAfor	5'-GCGAACAGATGCTGCATAGC
qNagArev	5'-CTTCAAGCATCGCATGACCG
qNagRfor	5'-TGATGCAAGCACAAGGCAAG
qNagRrev	5'-GCCGCCGTTTCAAAGCTAAT
qNagBfor	5'-ACCATTTGACGCAGAAACGC
qNagBrev	5'-CATGGAGTAGGCGTAGCGAG
EMSArcF	5'-GGCGGGGACCTCAGTTC
EMSArcR	5'-ACACTCCTTTTTAAGTGGGC
EMSArbF	5'-CTGCCATTTTTTTCGGCTC
EMSArbR	5'-TTTATGACCCCCTCCAAAG
EMSAaF	5'-CGTCAAGGATAAGTATGACG
EMSAaR	5'-CTGTAACCGTCACAATACC
nagBF	5'-CTCGCATCATACTGAGGACGC

TABLE S1. Continued from the previous page

nagBR	5’-TGATATCCCCCTATCTGGTCTAG
CD-F	5’-CAAGTATCAGATCTGGGAGACCACTAACATCCCATCACGCTGG
CD-R	5’- GTTAGCAGCCGGATCTACTAGTAAGCCGTGTATTGTGCCGCC
pT1NX-F	5’- CTAGTAGATCCGGCTGCTAAC
pT1NX-R	5’- GGTCTCCCAGATCTGATACTTG

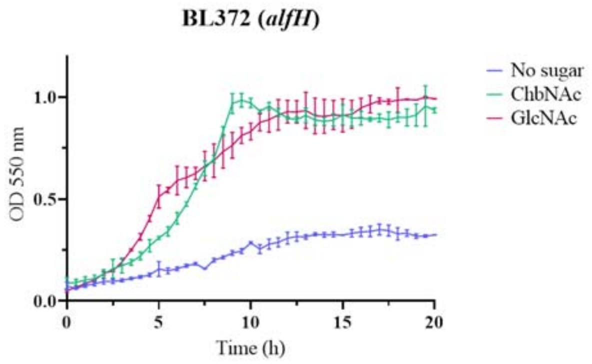

Supplementary Fig. S1. Growth curves of *Lacticaseibacillus paracasei* mutant strain BL372 (*alfH*) on MRS basal medium without carbon source (blue), with *N*,*N'*-diacetylchitobiose (ChbNAc) (green) or *N*-acetylglucosamine (GlcNAc) (magenta). Data presented are mean values based on at least three replicates. Error bars indicate standard deviations.

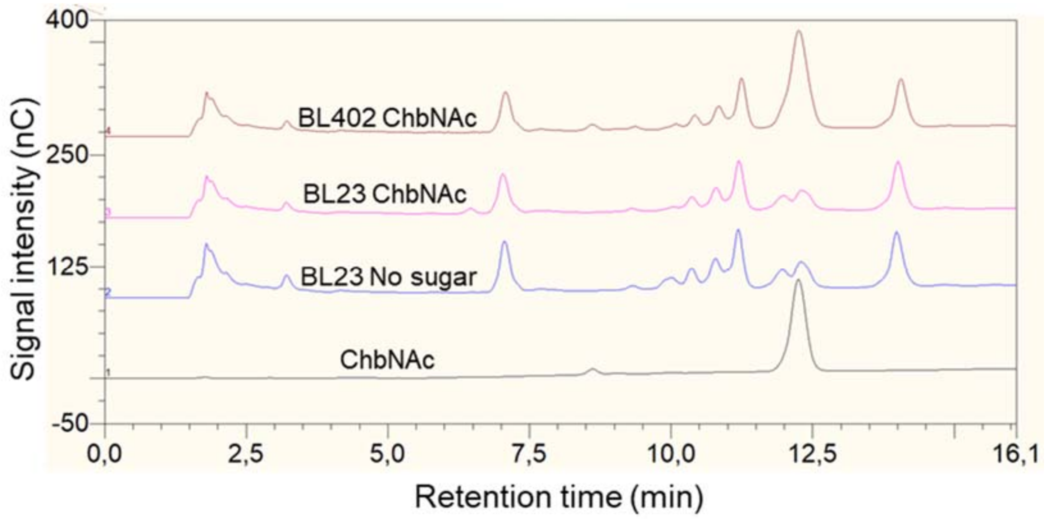

Supplementary Fig. S2. HPLC chromatograms (Dionex system) of the standard compound *N*,*N'*-diacetylchitobiose (ChbNAc) (0.2 mM) (chromatogram 1), and culture supernatants (diluted 20 times) from *Lacticaseibacillus paracasei* wild type BL23 grown without sugar (chromatogram 2) and with ChbNAc 4mM (chromatogram 3), and mutant BL402 (*chbC*) grown with ChbNAc 4mM (chromatogram 4). nC, nanoCoulomb.

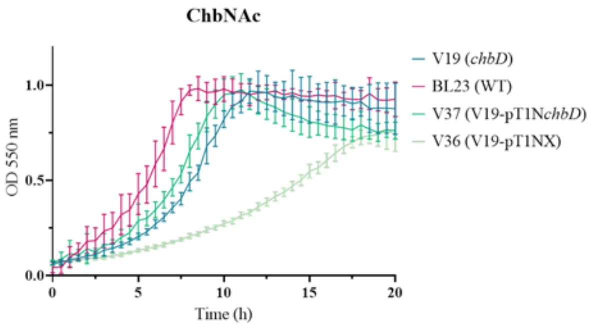

Supplementary Fig. S3. Growth curves of *Lacticaseibacillus paracasei* wild-type (WT) strain BL23 (magenta), mutant strains V19 (*chbD*) (teal), V37 (V19-pT1N*chbD*) (dark green) and V36 (V19-pT1NX) (light green) on MRS basal medium supplemented with 4mM *N*,*N*'-diacetylchitobiose (ChbNAc).

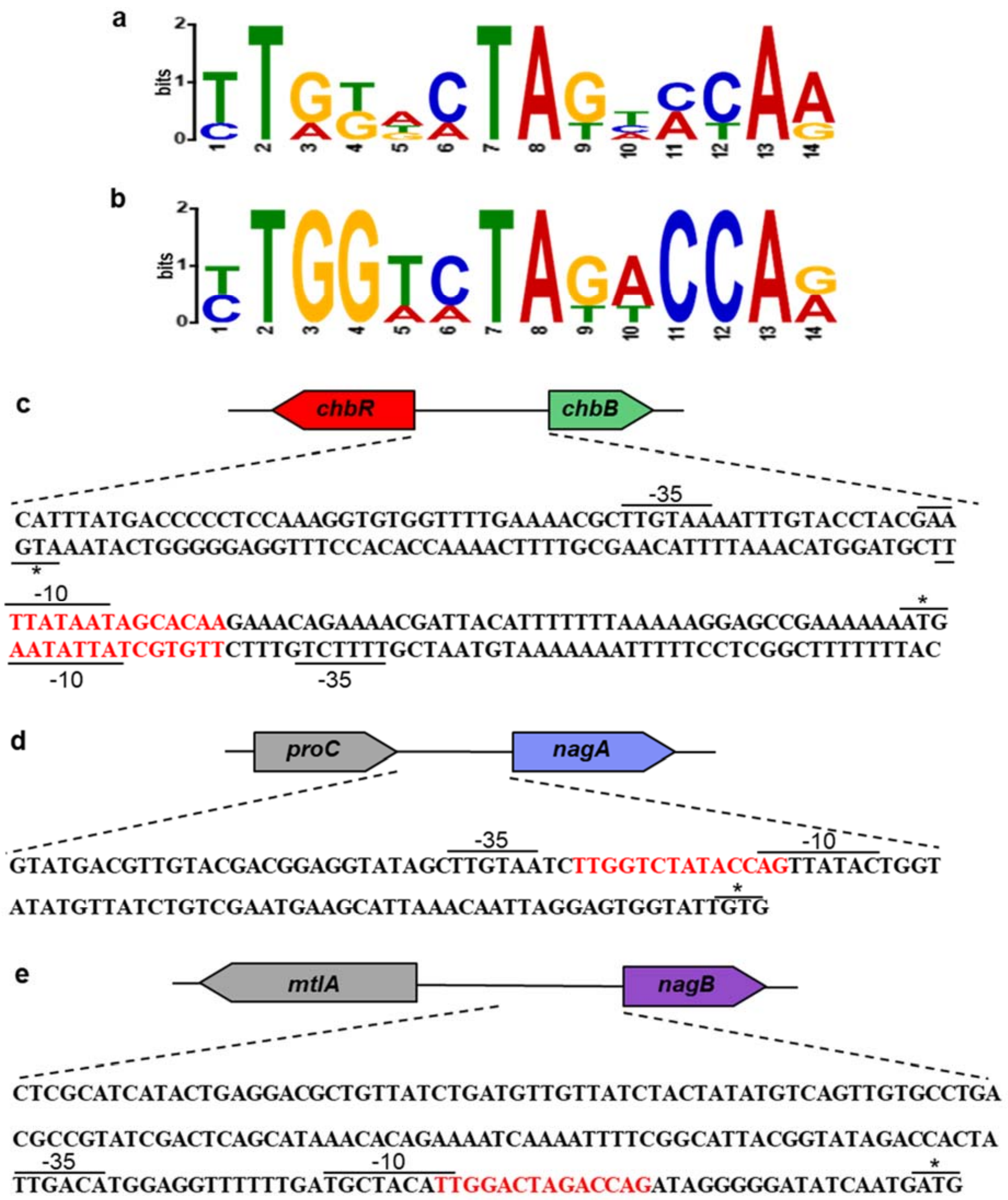

Supplementary Fig. S4. Identification of potential ChbR (a) and NagR (b) binding motifs analyzed by MEME (https://meme-suite.org) using the DNA sequences bound by ChbR: (c) and (e); and by NagR: (d) and (e). The sequences of the *chb* (c), *nagA* (d) and *nagB* (e) promoter regions are displayed. The start codons of *chbR*, *chbB, nagA* and *nagB* are marked with an asterisk. The -35 and -10 sequences of the putative promoters, analyzed by BPROM (http://www.softberry.com), are underlined. The putative ChbR and NagR binding sites are shown in red. In the *chb* promoter (c), plus and minus strands are depicted, showing the -35 and -10 sequences and ChbR binding sites for the divergently transcribed *chbR* and *chbB* genes.

a

B. subtilis nagP	AT**TGG**TA**TA**G**A**T**CA**CT
B. subtilis nagA	GC**TGG**TC**TA**G**A**T**CA**CT
S. coelicolor nagE1	AC**TGG**TC**TA**G**A**C**CA**CT
S. coelicolor nagB	TG**TGG**TT**TA**G**A**C**CA**AT
L. paracasei nagA	CT**TGG**TC**TA**T**A**C**CA**GT
L. paracasei nagB	AT**TGG**AC**TA**G**A**C**CA**GA

b

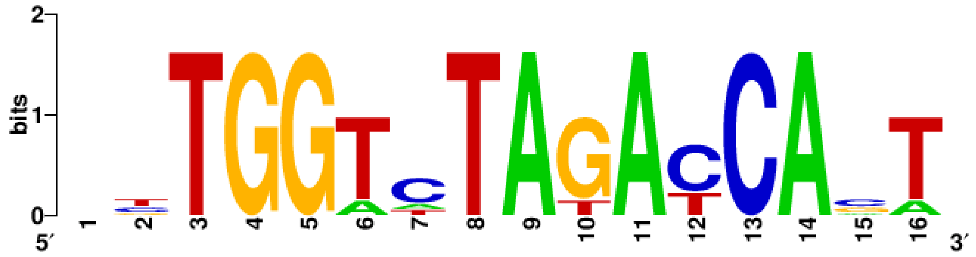

Supplementary Fig. S5. (a) Alignment of NagR binding sites from *Lacticaseibacillus paracasei nag* genes and from *Bacillus subtilis nagA* and *nagP* (GlcNAc-specific EIIBC component of the PTS) and *Streptomyces coelicolor nagB* and *nagE1* (EIIC component of a GlcNAc-specific PTS); (b) Sequence logo of nucleotide conservation of the binding sites for *L. paracasei* NagR, *B. subtilis* NagR and *S. coelicolor* DasR.
